# Networked Time Series Imputation via Position-aware Graph Enhanced Variational Autoencoders

**DOI:** 10.1145/3580305.3599444

**Published:** 2023-08-04

**Authors:** Dingsu Wang, Yuchen Yan, Ruizhong Qiu, Yada Zhu, Kaiyu Guan, Andrew Margenot, Hanghang Tong

**Affiliations:** University of Illinois at Urbana-Champaign, IL, USA; University of Illinois at Urbana-Champaign, IL, USA; University of Illinois at Urbana-Champaign, IL, USA; IBM Research, NY, USA; University of Illinois at Urbana-Champaign, IL, USA; University of Illinois at Urbana-Champaign, IL, USA; University of Illinois at Urbana-Champaign, IL, USA

**Keywords:** Networked time series, imputation, variational autoencoders, random walk with restart, node positional embeddings

## Abstract

Multivariate time series (MTS) imputation is a widely studied problem in recent years. Existing methods can be divided into two main groups, including (1) deep recurrent or generative models that primarily focus on time series features, and (2) graph neural networks (GNNs) based models that utilize the topological information from the inherent graph structure of MTS as relational inductive bias for imputation. Nevertheless, these methods either neglect topological information or assume the graph structure is fixed and accurately known. Thus, they fail to fully utilize the graph dynamics for precise imputation in more challenging MTS data such as *networked time series* (*NTS*), where the underlying graph is constantly changing and might have missing edges. In this paper, we propose a novel approach to overcome these limitations. First, we define the problem of imputation over NTS which contains missing values in both node time series features and graph structures. Then, we design a new model named PoGeVon which leverages variational autoencoder (VAE) to predict missing values over both node time series features and graph structures. In particular, we propose a new node position embedding based on random walk with restart (RWR) in the encoder with provable higher expressive power compared with message-passing based graph neural networks (GNNs). We further design a decoder with 3-stage predictions from the perspective of multi-task learning to impute missing values in both time series and graph structures reciprocally. Experiment results demonstrate the effectiveness of our model over baselines.

## 1 INTRODUCTION

Multivariate time series (MTS) data are common in many real-world applications, such as stock prediction [13, 71], traffic forecasting [43, 79, 80] and pandemic analysis [31, 52]. However, these data are often incomplete and contain missing values due to reasons such as market close or monitoring sensor/system failure. Predicting the missing values, which is referred to as the MTS imputation task, plays an important role in these real-world applications.

Recently, a large amount of approaches emerge for MTS imputation [17] in the literature. To name a few, BRITS [5] is built upon bidirectional recurrent modules and GAIN [77] is one of the earliest works that use adversarial training for the task. However, many of them ignore the available relational information within the data and thus are less effective to predict missing values compared to those considering both spatial and temporal information. In order to tackle this problem, some recent works utilize GNNs or other similar algorithms to assist the imputation over MTS data. GRIN [11] adopts a bidirectional recurrent model based on message passing neural networks [22]. They perform a one-step propagation of the hidden representations on the graph to capture the spatial dependencies within the MTS data. SPIN [48] is a follow-up method which solves the error accumulation problem of GRIN in highly sparse data. It introduces a new attention mechanism to capture spatial-temporal information through inter-node and intra-node attentions. By stacking several attention blocks, the model simulates a diffusion process and can handle data with high missing rates. Recently, NET^3^ [29] generalizes the setting and studies tensor time series data in which the underlying graph contains multiple modes. The authors utilize a tensor graph convolution network (GCNs) and a tensor recurrent neural network (RNNs) to handle the tensor graphs and time series respectively.

**Figure 1:**
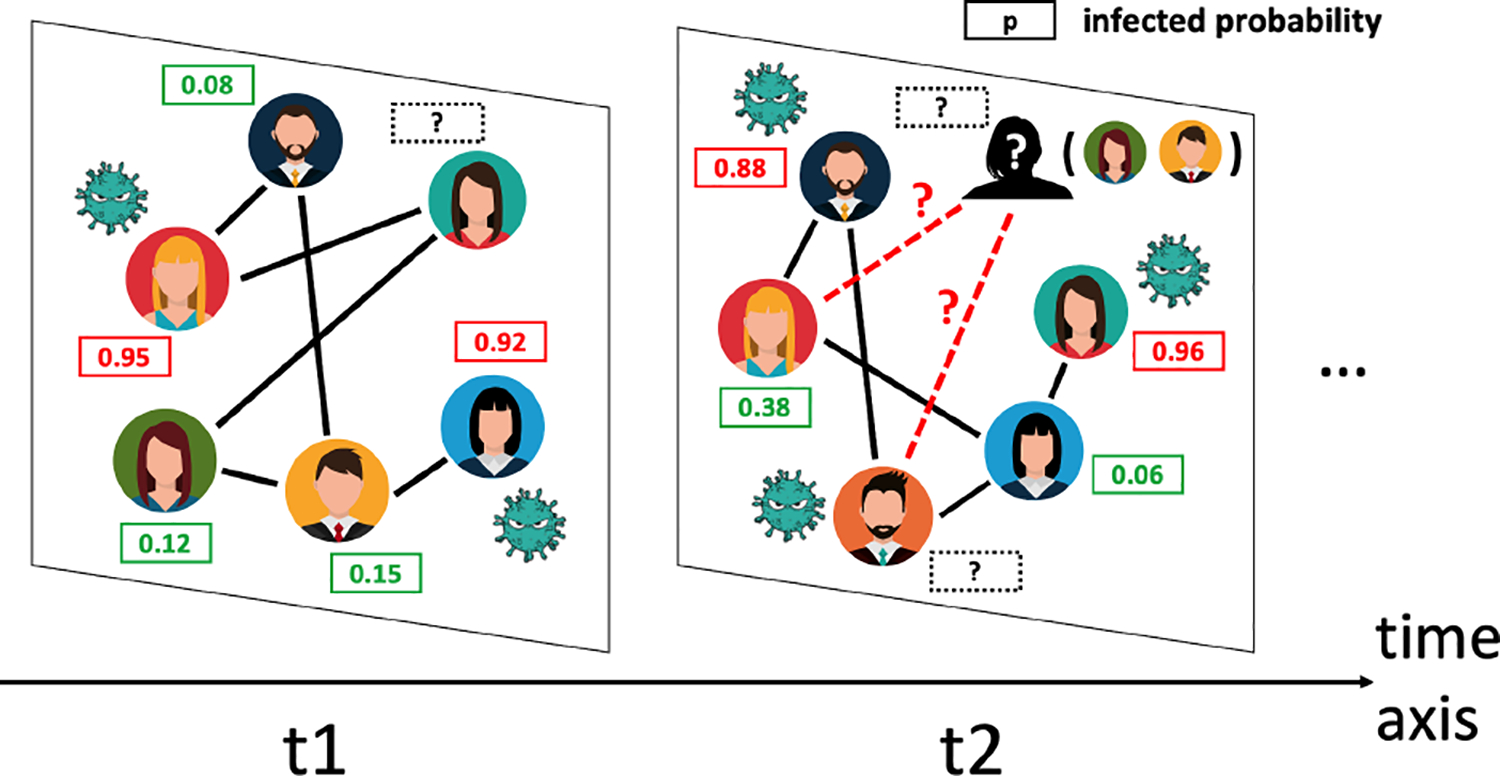
An illustrative example of an interaction network during the COVID-19 pandemic where some patients’ infection status might not be available and we have no access to whom these people interact with, which represents a networked time series (NTS) with both missing node features and missing edges.

Despite the strong empirical performance of these methods on the MTS imputation problem, they rely on the assumption that the underlying graph is fixed and accurately known. However, the graph structure of an MTS may constantly change over time in real-world scenarios. Take epidemiological studies as an example, during the evolution of a pandemic, individuals like human beings or animals may move around and thus the graph that models the spread of disease is dynamic. In literature, such time series data are referred to as *networked time series* (*NTS*)^1^ [29]. Given the nature of NTS data, the missing components can occur in both the node features and the graph structures (See an example in Figure 1), which makes NTS imputation an essentially harder problem compared to MTS imputation.

In this paper, we first formally define the problem of NTS imputation. We point out that the key challenges of this problem are twofold: (1) The graph that lies behind time series data is evolving constantly, and contains missing edges. Therefore, algorithms should capture the graph dynamics and at the same time be able to restore the lost structures. (2) The node feature time series also contains missing values, which requires the model to solve a general MTS imputation problem as well. To address these challenges, we formulate NTS imputation as a *multi-task learning* problem and propose a novel model named PoGeVon based on variational autoencoder (VAE) [35]. Our proposed model consists of two parts, including a recurrent encoder with node position embeddings based on random walk with restart (RWR) [60] and a decoder with 3-stage predictions. The *global* and *local* structural information obtained from RWR with respect to a set of anchor nodes provides useful node representations. Moreover, the 3-stage prediction module in the decoder is designed to impute missing features in time series and graph structures reciprocally: the first stage prediction fills the missing values for node features and then is used for the imputation over graph structures during the second stage, in return, the predicted graph structures are used in the third stage for node feature imputation. Finally, we replicate the VAE model in PoGeVon to handle the bidirectional dynamics in the NTS data. The main contributions of this paper can be summarized as:

- **Problem Definition.** To our best knowledge, we are the first to study the joint problem of MTS imputation and graph imputation over networked time series data.
- **Novel Algorithm and Analysis.** We propose a novel imputation model based on VAE, which consists of an encoder with RWR-based node position embeddings, and a decoder with 3-stage predictions. We provide theoretical analysis of the expressive power of our position embeddings compared with message-passing based temporal GNNs, as well as the benefit of multi-task learning approach for NTS imputation problem from the perspective of information bottleneck.
- **Empirical Evaluations.** We demonstrate the effectiveness of our method by outperforming powerful baselines for both MTS imputation and link prediction tasks on various real-world datasets.

The rest of the paper is organized as follows. Section 2 defines the imputation problem over NTS data. Section 3 presents the proposed PoGeVon model. Section 4 shows the experiment results. Related works and conclusions are given in Section 5 and Section 6 respectively.

## 2 PROBLEM DEFINITION

Table 1 lists main symbols and notations used throughout this paper. Calligraphic letters denote tensors or graphs (e.g., 𝓧,𝓖), bold uppercase letters are used for matrices (e.g., A), bold lowercase letters are for vectors (e.g., v). Uppercase letters (e.g., T) are used for scalars, and lowercase letters (e.g., i) are for indices. For matrices, we use A[i,j] to denote the value at the i-th row and j-th column.

We first present some necessary preliminaries and then introduce the networked time series imputation problem in this section.

Definition 2.1 (**Multivariate Time Series (MTS)**). A multivariate time series $X\in R^{T\times N\times D}$ is a sequence of observations: $\left\{{\text{X}}_{1},{\text{X}}_{2},\ldots ,{\text{X}}_{T}\right\}$, where each observation ${\text{X}}_{t}\in R^{N\times D}$ is a slice of 𝓧 at time step t that contains N entities with D features.

Definition 2.2 **(Networked Time Series (NTS))**. Networked time series is an extension of multivariate time series, in which a sequence of graphs $𝓖(𝓐,𝓧)=\left\{G_{1},G_{2},\ldots ,G_{T}\right\}$ *is given, and* 𝓐 *models the node interactions as time goes by. Each graph* $G_{t}$ *is represented as a weighted adjacency matrix* ${\text{A}}_{t}\in R^{N\times N}$ *with the node feature matrix* ${\text{X}}_{t}\in R^{N\times D}$.

Definition 2.3 **(Mask Tensor)**. *A binary mask tensor* $𝓜:\left\{M_{1},M_{2},\ldots ,M_{T}\right\}\in R^{T\times N\times D}$ *serves as the indicator of missing values in MTS data, in which the value* $M_{t}[i,j]$ *indicates the availability of each feature* j *of entity* i *at time step* $t\;:\;M_{t}[i,j]$ *being 0 or 1 indicates the corresponding feature is missing or observed*.

Given the nature of NTS data, its missing data can occur in two parts: (1) missing values in node feature time series, and (2) missing edges in graph structures. The former is similar to missing values in traditional MTS, while the latter is unique in NTS which demonstrates the underlying dynamics of a graph sequence. Therefore, we can also define mask tensor for graph adjacency sequence similar to Definition 2.3. We formally define the partially observed NTS data and NTS imputation problem as follows:

Definition 2.4 **(Partially Observed NTS).** *A partially observed NTS*: $𝓖(\tilde{𝓐},\tilde{𝓧})=\left\{{\tilde{G}}_{1},{\tilde{G}}_{2},\ldots ,{\tilde{G}}_{T}\right\}$ *consists of observed graph adjacency tensor* $\tilde{𝓐}$ *and observed node feature tensor* $\tilde{𝓧}$. *The value of* ${\tilde{A}}_{t}[i,j]$ *and* ${\tilde{X}}_{t}[i,j]$ *can be observed only if* $M_{t}^{A}[i,j]=1$ *and* $M_{t}^{X}[i,j]=1$ *where* ${𝓜}^{A}$ and ${𝓜}^{X}$ *are the mask tensors for graph adjacency structure and node features respectively*.

Problem 1 **(NTS Imputation)**.

**Given:** *A partially observed NTS with graph sequence* $𝓖(\tilde{𝓐},\tilde{𝓧})=\left\{{\tilde{G}}_{1},{\tilde{G}}_{2},\ldots ,{\tilde{G}}_{T}\right\};$

**Output:** The predicted graph adjacency tensor 𝓐 and the tensor 𝓧 *of node feature time series*.

*Note*. For clarity, we use node features and node time series interchangeably, and same for the graph adjacency imputations and missing edges/links predictions.

## 3 METHODOLOGY

In this section, we introduce our model named Position-aware Graph Enhanced Variational Autoencoders (PoGEVON) in detail. In order to predict the missing values in both the node features and the graph structures, we design a novel *variational autoencoder* (*VAE*), whose detailed architecture is shown in Figure 3. It consists of an encoder with node position embeddings based on *random walk with restart* (*RWR*), and a decoder with 3-stage predictions. We then replicate the VAE to handle bidirectional dynamics. We start in Subsection 3.1 to discuss the multi-task learning setting of NTS imputation problem, analyze its mutual benefit and implications to the encoder/decoder design. Then, we present the details of the proposed encoder (Subsection 3.2) and decoder (Subsection 3.3), followed by the training objective in Subsection 3.4 as well as the compelxity analysis in Subsection 3.5.

### 3.1 Multi-Task Learning Framework

**Figure 2:**
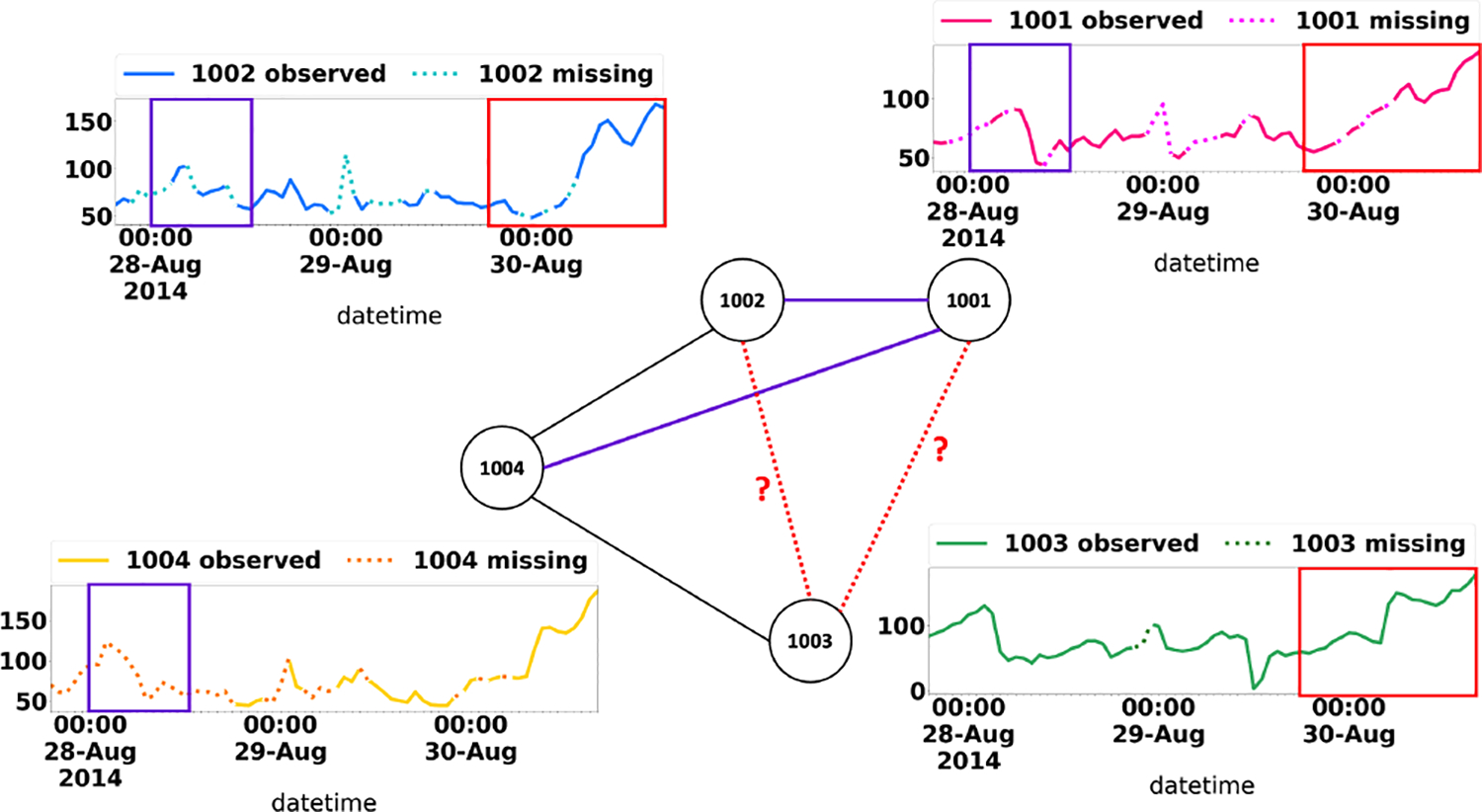
An illustrative example of mutual reinforcing effect between node feature imputation and graph structure imputation, based on 4 monitor stations in AQ36 dataset (See Section 4 for the details of the dataset). Correlation between three time series (1001, 1002, and 1003, indicated by three red boxes) helps impute the missing edges between them (the two red dashed lines). Meanwhile, the edges between 1001, 1002 and 1004 (the two purple lines) helps impute time series/node features by capturing the lagged correlation between them (the three purple boxes). Best viewed in color.

**Figure 3:**
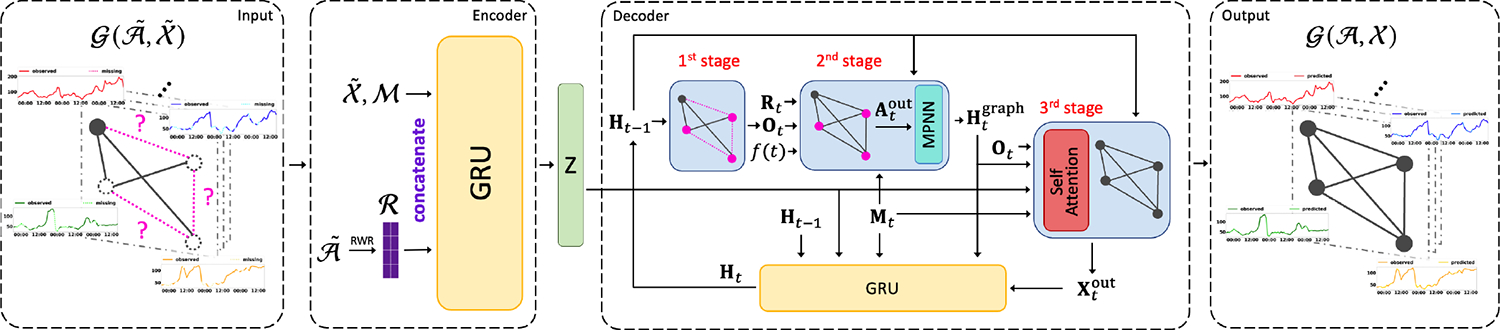
The model architecture of the proposed PoGeVon.

Because of the potential mutual benefit of predicting missing node features and edges, it is natural to formulate the NTS imputation as a multi-task learning problem which consists of the *imputation* task for node time series and the *link prediction* task for graph structures. Let us analyze the benefit of modeling NTS imputation as a multi-task learning problem from the perspective of *information bottleneck* in unsupervised representation learning [1, 59], and formulate the objective of NTS imputation as:

(1) $$\text{max}\left[I(\tilde{𝓐},\tilde{𝓧};z)-\beta I\left(z;{\tilde{𝓖}}_{t:t+\Delta t}\right)\right]$$

where z is the latent representation, I(·;·) is the mutual information, ${\tilde{𝓖}}_{t:t+\Delta t}$ is the data sample which represents a sliding window of NTS data and β is the Lagrange multiplier. This formulation closely relates to the objective of a β-VAE [1, 25]. Here, the second term $\beta I\left(z;{\tilde{𝓖}}_{t:t+\Delta t}\right)$ in Eq. (1) constraints the amount of identity information of each data sample that can transmit through the latent representation z. In β-VAE, this is upper bounded by minimizing the Kullback–Leibler divergence $\beta \cdot KL\left[q_{\theta }(z|X)\|p(z)\right]$ [3]. The first term $I(\tilde{𝓐},\tilde{𝓧};z)$ in Eq. (1) represents the reconstruction task of VAE which can be decomposed as [28]:

(2) $$I(\tilde{𝓐},\tilde{𝓧};z)=I(\tilde{𝓐};z)+I(\tilde{𝓧};z)-I(\tilde{𝓐};\tilde{𝓧};z)$$

where $I(\tilde{𝓐},\tilde{𝓧};z)$ represents the mutual information between the partially observed NTS $\tilde{𝓖}$ (i.e., the joint distribution of $\tilde{𝓐}$ and $\tilde{𝓧}$) and z, while $I(\tilde{𝓐};\tilde{𝓧};z)$ is the High-order Mutual Information [28, 49], which measures the shared information among multiple different random variables (i.e., $\tilde{𝓐},\;\tilde{𝓧}$, and z). It is worthy noting that when $\tilde{𝓐}$ and $\tilde{𝓧}$ are independent from each other (even given z), we have:

(3) $$I\left(\tilde{𝓐},\tilde{𝓧};z\right)=H\left(\tilde{𝓐},\tilde{𝓧}\right)-H\left(\tilde{𝓐},\tilde{𝓧}|z\right)\;=H\left(\tilde{𝓐}\right)+H\left(\tilde{𝓧}\right)-H\left(\tilde{𝓐}|z\right)-H\left(\tilde{𝓧}|z\right)=I\left(\tilde{𝓐};z\right)+I\left(\tilde{𝓧};z\right)$$

where H(⋅) is the entropy. Compared with Eq. (2), it is clear that $I(\tilde{𝓐};\tilde{𝓧};z)$ now equals to 0. Under such circumstances, i.e., no correlation exists between features of any adjacent node pairs, the objective in Eq. (1) becomes modeling time series features and graph structures independently. However, in reality, this is often not the case. Figure 2 demonstrates an illustrative example from the AQ36 dataset [82] in which NTS imputation problem occurs when monitor stations fail due to system errors and lose data as well as connections with each other. To maximize Eq. (2), we further decompose $I(\tilde{𝓐};\tilde{𝓧};z)$:

(4) $$I(\tilde{𝓐};\tilde{𝓧};z)=I(\tilde{𝓐};z)-I(\tilde{𝓐};z|\tilde{𝓧})=I(\tilde{𝓧};z)-I(\tilde{𝓧};z|\tilde{𝓐})$$

where the second equation holds because of symmetry [75]. Combining Eq. (2) and Eq. (4), we can derive the objective term for the decoder as:

(5) $$2\cdot I(\tilde{𝓐},\tilde{𝓧};z)=\underset{\text{VAE}}{\underbrace{I(\tilde{𝓐};z)+I(\tilde{𝓧};z)}}+\underset{\text{Conditional}\;\text{VAE}}{\underbrace{I(\tilde{𝓧};z|\tilde{𝓐})+I(\tilde{𝓐};z|\tilde{𝓧})}}$$

where the first two terms can be bounded by the objective for VAE decoder as in [1]. The last two terms represent the objective of conditional VAE (CVAE) since $I(\tilde{𝓧};z|\tilde{𝓐})=H(\tilde{𝓧}|\tilde{𝓐})-H(\tilde{𝓧}|\tilde{𝓐},z)$. The first term $H(\tilde{𝓧}|\tilde{𝓐})$ on the right hand can be dropped because it is independent from our model, and maximizing the second term $-H(\tilde{𝓧}|\tilde{𝓐},z)$ is essentially the same as optimizing the decoder of CVAE with objective $\text{max}\;p(\tilde{𝓧}|\tilde{𝓐})$. Similar analysis applies to $I(\tilde{𝓐};z|\tilde{𝓧})$. Eq. (5) provies an important insight: we can use $\tilde{𝓐}$ and $\tilde{𝓧}$ as the conditions for each other’s predictions since imputation over one of them might be instructive for the other.

To summarize, our analysis reveals that (1) when the features of adjacent nodes are uncorrelated, we can impute the node time series and graph adjacency independently (Eq. (3)); however, (2) in real applications, node features and graph structure are often correlated (e.g., Figure 2), and in such a scenario, there might be a mutual reinforcing effect between node feature imputation and graph adjacency imputation (Eq. (4)). Our analysis also provides novel and critical clues that can guide the design of the encoder-decoder framework for learning datasets with multi-modality such as NTS. For the encoder, Eq. (5) suggests that the latent representation z (i.e., the output of the encoder) should encode both the graph adjacency information and the node feature information (i.e., the VAE part of Eq. (5)) as well as the mutual interaction between them (i.e., the CVAE part of Eq. (5)). For the decoder, we will present a three-stage prediction method so that the (imputed) graph structures and the (imputed) node features can be used as each other’s condition respectively (i.e., the CVAE part of Eq. (5)).

### 3.2 Encoder

The encoder aims to encode both the structural and the dynamic information of NTS data. Existing message-passing based GNNs typically only capture the *local* information from close neighbors. However, long-distance information between nodes is important in NTS data since the graph is constantly evolving and interactions between nodes can occur at any time step. Therefore, to capture this long-distance global information, we propose using position embeddings with random walk with restart (RWR) [20, 60, 73, 74].

#### 3.2.1 RWR-based Position Embeddings.

For a graph $G_{t}$ at time step t, the relative position vector for all nodes w.r.t. one anchor node i is computed by RWR as follows:

(6) $$r_{t,i}=(1-c){\overset{ˆ}{A}}_{t}r_{t,i}+ce_{i}$$

where ${\overset{ˆ}{A}}_{t}={\left(D_{t}^{-1}A_{t}\right)}^{⊤}$ is the normalized adjacency matrix of $G_{t},\;e_{i}\in R^{N}$ is a one-hot vector which only contains nonzero value at position i and c is the restart probability. After reaching the stationary distribution, we concatenate the position scores $r_{t,i}\in R^{N}$ of all the anchor nodes as the final position embeddings $R_{t}\in R^{N\times N}$, where N is the number of nodes.

We next prove the expressive power of RWR-based position embeddings with following proposition and theorem.

Proposition 3.1. *Random walk with restarts (RWR) captures information from close neighbors (local) and long-distance neighbors (global) in graph learning*.

Proof. See Appendix. □

The benefit of RWR-based position embeddings in temporal graphs is summarized in Theorem 3.2 from the perspective of message-passing based temporal graph networks (TGN) [55], which is a general class of GNNs designed for handling temporal graphs. It contains two main components: *memory* (through RNNs) for capturing the dynamics of each node; *aggregate and update* (through GNNs) for gathering topological information from neighbors.

Theorem 3.2. *Given a temporal graph* 𝓖, TGN with RWR-based node position embeddings $g_{\theta }$ has more expressive power than regular *TGN* $f_{\theta }$ *in node representation learning:* D(g(u),g(v))≥D(f(u),f(v)) *where* D(⋅,⋅) *measures the expressiveness by counting the distinguishable node pairs* (u,v) *in* 𝓖 *based on node representations*.

Proof. See Appendix. □

Finally, to capture the dynamic information in NTS data, we use a 2-layer gated recurrent unit (GRU) [10] as the encoder to model $q_{\theta }(z|\tilde{𝓧},𝓜,𝓡)$, where z is the latent representation and $𝓡=\left\{R_{1},\ldots ,R_{T}\right\}$ is the tensor of node position embeddings. For each ${\text{R}}_{t}$, instead of treating all the nodes as anchor nodes, usually only a small subset of anchor nodes |S|=L would be sufficient to distinguish nodes from each other in practice [78]. Masks 𝓜 and position embeddings 𝓡 are concatenated with the input $\tilde{𝓧}$ at each time step before feeding into the GRU.

### 3.3 Decoder

We design the decoder as a GRU with 3-stage predictions. We use ${\text{H}}_{t}$ to denote node embedding matrix at time step t and 𝓗 to denote node embedding tensor. Based on the analysis in Section 3.1, we model the complementary relation between feature imputation $p_{\phi }(\tilde{𝓧}|\tilde{𝓐},𝓜,z)$ and network imputation $p_{\gamma }(\tilde{𝓐}|𝓜,𝓡,z)$ at different prediction stages in the decoder as follows.

#### 3.3.1 First-stage Feature Prediction.

In the first stage, we use a linear layer to generate an initial prediction of the missing values in the time series:

(7) $${\overset{ˆ}{Y}}_{1,t}=\text{Linear}\left(H_{t-1}\right)$$

where $H_{t-1}$ is the hidden representation of each node from the previous time step and $H_{0}$ is sampled from a normal distribution $N\left(0,1/\sqrt{d_{h}}\right)$ where $d_{h}$ is the hidden dimension. Similar to [11], we then use a filler operator to replace the missing values in the input ${\tilde{\text{X}}}_{t}$ with ${\overset{ˆ}{\text{Y}}}_{1,t}$ to get the first-stage output ${\text{O}}_{t}$:

(8) $$O_{t}=M_{t}⊙{\tilde{X}}_{t}+\left(1-M_{t}\right)⊙{\overset{ˆ}{Y}}_{1,t}$$

#### 3.3.2 Second-stage Link Prediction.

Our second-stage prediction imputes the missing weighted edges within graphs. $O_{t}$ is used with the mask $M_{t}$, the position embedding $R_{t}$ and $H_{t-1}$ to get the embeddings of all nodes at timestep t through a linear layer:

(9) $$U_{t}=\text{Linear}\left(O_{t}\left\|M_{t}\right\|R_{t}\|H_{t-1}\right)$$

where ‖ is concatenation. We directly use the hidden states from previous time step $H_{t-1}$ as the embeddings for those missing nodes since no new features or graph structures of them are available at time step t. In NTS, observations are usually obtained by irregular sampling and the imputation problem over them can occur at any future step in real world problems. Being able to handle such uncertainty and forecasting unseen graph structure/time series data in the future time step are two key characteristics of an NTS imputation model. Therefore, in order to capture the dynamics between different timestamps and enhance the expressiveness of PoGeVon, we also encode the time information with learnable Fourier features based on Bochner’s theorem [68, 69], whose properties are summarized in Proposition 3.3, as follows:

(10) $$f(t)=\sqrt{\frac{1}{k}}\left[\text{cos}\;\left(w_{1}t\right),\text{sin}\;\left(w_{1}t\right),\ldots ,\text{cos}\;\left(w_{k}t\right),\text{sin}\;\left(w_{k}t\right)\right]$$

where $w_{1},\ldots ,w_{k}$ are learnable parameters.

Proposition 3.3. *Time encoding function* f(t) *is invariant to time rescaling and generalizes to any future unseen timestamps*.

Proof. See [33, 69]. □

Then, we concatenate node embeddings with time encodings through broadcasting as the input of a two-layer multi-layer perceptron (MLP) to predict the missing edges:

(11) $$A_{t}^{\text{out}}=\text{MLP}\left(U_{t}\left\|H_{t-1}\right\|f(t)\right)$$

The next step is to enhance node embeddings with updated graph structures. The general class of message-passing neural networks (MPNNs) [22] is used similar to the *aggregate and update* step in TGN to capture the graph topological information, which can be defined as:

(12) $$H_{t}^{\text{graph}}=\text{MPNN}\left(U_{t},A_{t}^{\text{out}}\right)$$

whose detailed design can be found in Appendix.

#### 3.3.3 Third-stage Feature Prediction.

In the third-stage prediction, we utilize the structural information $H_{t}^{\text{graph}}$ to make a fine-grained imputation again over node features time series. Aiming to enhance the semantics of the node representations, we apply a self attention layer [61] to capture cross-node information in our third-stage prediction, which helps to encode richer node interaction information that is not captured in $H_{t}^{\text{graph}}$. The latent node representations Z, previous hidden state $H_{t-1}$, the structural representation $H_{t}^{\text{graph}}$ and the first stage output $O_{t}$ as well as the masks $M_{t}$ are all concatenated and processed by a self attention layer with an MLP to get the final output imputation representations:

(13) $$H_{t}^{\text{out}}=\text{MLP}\left(\text{Attn}\left(Z\left\|H_{t-1}\right\|H_{t}^{\text{graph}}\left\|O_{t}\right\|M_{t}\right)\right)$$

Then a two-layer MLP is used for the third-stage prediction:

(14) $${\overset{ˆ}{Y}}_{2,t}=\text{MLP}\left(H_{t}^{\text{out}}\left\|H_{t-1}\right\|H_{t}^{\text{graph}}\right)$$

A filler operator similar to Eq. (8) is applied to get the imputation output ${\text{X}}_{t}^{\text{out}}$ from ${\overset{ˆ}{Y}}_{2,t}$. Finally, a single layer GRU is used similar to the *memory* step in TGN to update hidden representations based on the latent node representation Z, the output of second-stage $X_{t}^{\text{out}}$, the mask $M_{t}$ and the structural representation $H_{t}^{𝓐}$ for each node and move on to the next time step:

(15) $$H_{t}=\text{GRU}\left(Z\left\|X_{t}^{\text{out}}\right\|M_{t}\|H_{t}^{\text{graph}}\right)$$

#### 3.3.4 Bidirectional Model.

Similar to [11], we extend our VAE model to bidirectional by replicating the architecture to handle both the forward and backward sequences. An MLP is used over the output hidden representations from these two VAEs to produce the final imputation output $\overset{ˆ}{𝒴}$:

(16) $$\overset{ˆ}{𝓨}=\text{MLP}\left({𝓗}_{f}^{\text{out}}\left\|{𝓗}_{b}^{\text{out}}\right\|{𝓗}_{f}^{\text{graph}}\left\|{𝓗}_{b}^{\text{graph}}\right\|{𝓗}_{f}\|{𝓗}_{b}\right)$$

where ${𝓗}^{\text{out}}$ is the tensor of imputation representations from the final stage prediction, f and b stand for forward and backward directions respectively. Algorithm 1 summarizes the detailed workflow of the proposed PoGeVon.

### 3.4 Objective and Training

The *Evidence Lower Bound* (*ELBO*) objective function of a vanilla conditional VAE [12, 14] over missing data imputations can be defined as:

(17) $$\text{ELBO}\left(\theta ,\phi \right)=E_{q}\left[\text{log}\;p_{\phi }\left(\tilde{𝓧}|z,𝓜\right)\right]-KL\left[q_{\theta }\left(z|\tilde{𝓧},𝓜\right)||p_{\phi }\left(z\right)\right]\leq \text{log}\;p_{\phi }\left(\tilde{𝓧}|𝓜\right)$$

**Table:** 

**Algorithm 1** PoGeVon: Position-aware Graph Enhanced Variational Autoencoders	
**Input:** A partially observed NTS: $𝓖(\tilde{𝓐},\tilde{𝓧})=\left\{{\tilde{G}}_{1},{\tilde{G}}_{2},\ldots ,{\tilde{G}}_{T}\right\}$.	
**Output:** The predicted tensor 𝓧 of node feature time series and the predicted graph adjacency tensor 𝓐.	
1:	Generate node position embeddings 𝓡 based on Eq. (6).
2:	**for** e=1,2,3,…, num_epochs **do**
3:	Encode ${\tilde{𝓧}}_{f}$, ${𝓜}_{f}$, ${𝓡}_{f}$ to get $z_{f}$ based on Section 3.2.
4:	**for** t=1,2,3,…,T (forward direction) **do**
5:	Perform first-stage decoding based on Section 3.3.1.
6:	Perform second-stage decoding based on Section 3.3.2.
7:	Perform third-stage decoding based on Section 3.3.3.
8:	**end for**
9:	Encode ${\tilde{𝓧}}_{b}$, ${𝓜}_{b}$, ${𝓡}_{b}$ to get $z_{b}$ based on Section 3.2.
10:	**for** t=T,T−1,…,1 (backward direction) **do**
11:	Perform first-stage decoding based on Section 3.3.1.
12:	Perform Second-stage decoding based on Section 3.3.2.
13:	Perform third-stage decoding based on Section 3.3.3.
14:	**end for**
15:	Generate final outputs $\hat{𝓨}$ based on Eq. (16).
16:	Update parameters θ, γ, ϕ by optimizing the loss in Eq. (19).
17:	**end for**
18:	Obtain the predicted tensor 𝓧 of node feature time series based on Eq. (8) by replacing missing values in $\hat{𝓧}$ with $\hat{𝓨}$.
19:	Obtain the predicted graph adjacency tensor 𝓐 based on Eq. (8) by replacing missing values in $\hat{𝓐}$ with ${𝓐}^{\text{out}}$.
20:	**return** the predicted tensor time series 𝓧 and the predicted tensor of graph adjacency 𝓐.

Our goal is to learn a good generative model of both the observed multivariate node feature time series $\tilde{𝓧}$ and the observed graph adjacency $\tilde{𝓐}$. Thus, we can treat the position embeddings 𝓡 as an extra condition in addition to the mask 𝓜 similar to [26]. This is because, 𝓜 and 𝓡 are auxiliary covariates, and are given or can be generated through deterministic functions based on $\tilde{𝓐}$ and $\tilde{𝓧}$ respectively. Therefore, it is more natural to maximize $\text{log}\;\;p(\tilde{𝓧},\tilde{𝓐}|𝓜,𝓡)$ as our objective, which is summarized in the following lemma.

Lemma 3.4. *Under the condition that* 𝓜 *and* 𝓡 *are jointly independent of the prior* p(z):p(z)=p(z∣𝓜,𝓡), *the new ELBO objective of the proposed PoGeVon for the NTS imputation problem is:*

(18) $$ELBO^{new}\left(\theta ,\gamma ,\phi \right)=E_{q}\left[\text{log}\;p_{\phi }\left(\tilde{𝓧}|\tilde{𝓐},𝓜,z\right)\right]+E_{q}\left[\text{log}\;p_{\gamma }\left(\tilde{𝓐}|𝓜,𝓡,z\right)-KL\left[q_{\theta }\left(z|\tilde{𝓧},𝓜,𝓡\right)\|p_{\phi }\left(z\right)\right]\right]$$

*where* γ *denotes parameters of the link prediction module*.

Proof. The derivation of ${\text{ELBO}}^{\text{new}}$ can be formulated as:

$$\text{log}\;p\left(\tilde{𝓧},\tilde{𝓐}|𝓜,𝓡\right)\;=\text{log}\int \;p\left(\tilde{𝓧},\tilde{𝓐}|𝓜,𝓡,z\right)p\left(z\right)dz=\text{log}\;\int \;p\left(\tilde{𝓧}|\tilde{𝓐},𝓜,𝓡,z\right)p\left(\tilde{𝓐}|𝓜,𝓡,z\right)p\left(z\right)dz$$

since the node position embedding 𝓡 can be generated from the observed graph adjacency $\tilde{𝓐}$,

$$=\text{log}\;\int \;p\left(\tilde{𝓧}|\tilde{𝓐},𝓜,z\right)p\left(\tilde{𝓐}|𝓜,𝓡,z\right)p\left(z\right)\frac{q\left(z|\tilde{𝓧},𝓜,𝓡\right)}{q\left(z|\tilde{X},𝓜,𝓡\right)}dz\;=\text{log}\;E_{q}\left[p\left(\tilde{𝓧}|\tilde{𝓐},𝓜,z\right)p\left(\tilde{𝓐}|𝓜,𝓡,z\right)\frac{p\left(z\right)}{q\left(z|\tilde{𝓧},𝓜,𝓡\right)}\right]\;\geq E_{q}\left[\text{log}\;p\left(\tilde{𝓧}|\tilde{𝓐},𝓜,z\right)]+E_{q}[\text{log}\;p\left(\tilde{𝓐}|𝓜,𝓡,z\right)\right]-KL[q\left(z|\tilde{X},𝓜,𝓡\right)||p\left(z\right)]$$

□

This lemma generalizes the ELBO in Eq. (17) to the multi-task learning setting which ensures the learning objective of the proposed PoGeVon is consistent with our analysis in Section 3.1. That is, ELBO^new^ corresponds to Eq. (1) by modeling dependencies between the observed node time series 𝓧 and observed graph adjacency 𝓐 similar to Eq. (5).

We use a similar strategy as in [12, 51] to maximize ELBO^new^ by training our model over observed data and infer missing ones based on $p(𝓧|\tilde{𝓧})\approx \int \;p(𝓧|z)q(z|\tilde{𝓧})dz$. We (1) use the mean absolute error (MAE) as the error function for the feature imputation and (2) use the Frobenius norm between the predicted adjacency matrices and the observed adjacency matrices as the link prediction loss. The model is trained by minimizing the following loss function which is composed of errors of all three stages:

(19) $$ℒ=\underset{{\text{First}\;\text{and}\;\text{third}\;\text{terms}\;\text{in}\;\text{ELBO}}^{new}}{\underset{︸}{L\left({\hat{𝒴}}_{t:t+\Delta t},{\tilde{𝓧}}_{t:t+\Delta t},{𝓜}_{t:t+\Delta t}\right)+\beta \cdot KL_{f}+\beta \cdot KL_{b}}}+\underset{\text{Error}\;\text{for}\;\text{the}\;1^{\text{st}}\;\text{stage}\;\text{prediction}}{\underset{︸}{L\left(O_{f,t:t+\Delta t},{\tilde{𝓧}}_{t:t+\Delta t},{𝓜}_{t:t+\Delta t}\right)+L\left(O_{b,t:t+\Delta t},{\tilde{𝓧}}_{t:t+\Delta t},{𝓜}_{t:t+\Delta t}\right)}}+\underset{\text{Error}\;\text{for}\;\text{the}\;2^{\text{nd}}\text{stage}\;\text{prediction}(\text{i.e}.,\;\text{second}\;\text{term}\;\text{in}\;\text{ELB}{\text{O}}^{\text{new}})}{\underset{︸}{\gamma \cdot {\left\|{\tilde{𝓐}}_{f,t:t+\Delta t}-{𝓐}_{f,t:t+\Delta t}^{\text{out}}\right\|}_{F}+\gamma \cdot {\left\|{\tilde{𝓐}}_{b,t:t+\Delta t}-{𝓐}_{b,t:t+\Delta t}^{\text{out}}\right\|}_{F}}}+\underset{\text{Error}\;\text{for}\;\text{the}\;3^{\text{rd}}\text{stage}\;\text{prediction}}{\underset{︸}{L\left({𝓧}_{f,:t:t+\Delta t}^{\text{out}},{\tilde{𝓧}}_{t:t+\Delta t},{𝓜}_{t:t+\Delta t}\right)+L\left({𝓧}_{b,t:t+\Delta t}^{\text{out}},{\tilde{𝓧}}_{t:t+\Delta t},{𝓜}_{t:t+\Delta t}\right)}}$$

where β is the weight for KL divergence similar to [25] and γ is the weight for the 2^nd^ stage prediction. The element wise error function $L\left({𝓧}^{\text{pred}},{𝓧}^{\text{label}},𝓜\right)$ outputs the average error by calculating the inner product between mask tensor 𝓜 and $\left|{𝓧}^{\text{label}}-{𝓧}^{\text{pred}}\right|$. The loss ℒ is optimized through each sample in the dataset which is a sliding window (t:t+Δt) of NTS data (i.e., $\left.{\tilde{𝓖}}_{t:t+\Delta t}\right)$.

### 3.5 Complexity Analysis

The computational complexity of PoGeVon can be analyzed through the following aspects. First, calculating the position embedding 𝓡 has the complexity $O\left(T\cdot \overset{‾}{E}\cdot \text{log}\;\frac{1}{\epsilon }\right)$ [62] where $\overset{‾}{E}$ is the average number of edges and ϵ is the absolute error bound for the power iteration of RWR. Second, with a standard bidirectional VAE based on GRU, MPNN increases the complexity by $O(\overset{‾}{E})$ with sparse matrix multiplications at each time step. Third, the self-attention used in the third-stage decoder has the complexity $O\left(N^{2}\right)$. There are several ways to reduce the overall time complexity. For example, most of the computations can be parallelized. One computational bottleneck lies in the computation of self-attention. The existing techniques for efficient attentions [58] can be readily applied in the proposed PoGeVon, such as Linformer [64] which uses low-rank projections to make the cost of the attention mechanism O(N) and Reformer [38] which applies locality sensitive hashing to reduce the complexity of attention to O(N⋅logN).

## 4 EXPERIMENT

We apply the proposed PoGeVon to the networked time series imputation task, and evaluate it in the following aspects:

- Q1. How effective is PoGeVon for networked time series imputation?
- Q2. To what extent does our method benefit from different components of the model?

### 4.1 Experimental Setup

#### 4.1.1 Datasets.

We evaluate the proposed PoGeVon model on five real-world datasets, and the statistics of all the datasets are listed in Table 2.

- **COVID-19:** A dataset of COVID-19 infection cases and deaths in 50 states in USA from 01/21/2020 to 12/31/2020 [32]. Similar to [31], we choose infection cases of states as the time series data X and use mobility of people across different states to model the spatial relationship A between them. Then, we apply a Radial Basis Function (RBF) $f(u,v,t)=\text{exp}\;\left(-\frac{{\left\|x_{t}^{u}-x_{t}^{v}\right\|}^{2}}{2\sigma^{2}}\right)$ [8] to capture the dynamics and generate the graph sequence. Finally, we simulate the missing edges in the NTS imputation problem by masking edges when one of its end nodes contains missing features. Specifically, an edge weight $w_{t}^{u,v}$ between nodes u and v at time t can be defined as:
(20) $$w_{t}^{u,v}=\{\begin{array}{ll} w^{u,v} & \text{if}\;\text{A}\left[u,v\right]\neq 0\;\text{and}\;f\left(u,v,t\right)>k\\ & \text{and}\;m_{t}^{u}=1,m_{t}^{v}=1.\\ 0 & \text{otherwise}. \end{array}$$
  where k is the positive threshold for graph dynamics and we choose k=0.3 for COVID-19 dataset. We randomly mask out 25% of the node features in this dataset, and split the time axis to 70% for training, 10% for validation and 20% for test respectively.
- **AQ36:** A dataset of AQI values of different air pollutants collected from various monitor stations over 43 cities in China [82]. Following [5, 11], we use the reduced version of the dataset which contains 36 nodes (**AQ36**) and pick the last four months as the test data. To construct the static graph G(A,X), we use the thresholded Gaussian kernel from [57] to get the pairwise distances A[u,v] between stations u and v as the edge weight. The graph sequence is constructed using the similar method as Eq. (20) over normalized time series features and the threshold k is set to 0.8. We use the same mask setting as [76] which simulates the true missing data distribution.
- **PeMS-BA/PeMS-LA/PeMS-SD** Three datasets contain traffic statistics based on the Caltrans Performance Measurement System (PeMS) [6], which cover the freeway system in major areas of California. We collect 5-minute interval traffic flow data from 3 different stations 4,7 and 11 between 01/01/2022 and 03/31/2022, which represent the traffic information from Bay Area, Los Angeles and San Diego respectively. For each dataset, we pick 64 sensors with the largest feature variance, and use their latitude and longitude values to calculate pairwise distances to build the static graph. We only keep edges with weight within certain threshold, and we use 15 miles for **PeMS-BA/PeMS-LA** and 10 miles for **PeMS-SD**. The graph sequence is constructed using the similar method as the AQ36 dataset, and the threshold k is set to 0.8. We use similar masking settings as COVID-19 dataset.

The missing rate of AQ36’s time series features is about 13.24%, while for COVID-19 dataset and all the traffic datasets, the time series features have 25% missing values. Based on Eq. (20), the missing rates of edges for AQ36 is 28.06%, for COVID-19 is 43.23%, and for PEMS-BA/PEMS-LA/PEMS-SD are 43.75%/43.74%/43.71% respectively.

To be consistent with the dataset settings in previous works such as GRIN [11], we use the following window length to train the models: (i) 14 for COVID-19 dataset which corresponds to 2 weeks, (ii) 36 for AQ36 dataset which corresponds to 1.5 days and (iii) 24 for all the traffic datasets which corresponds to 2 hours of data.

#### 4.1.2 Baselines.

We compare the proposed PoGeVon model with following baselines for the time series imputation task. All the methods are trained with NVIDIA Tesla V100 SXM2 GPU.

1. **Mean**. Impute with node level feature average along the sequence.
2. **Matrix Factorization (MF)**. Matrix factorization of the incomplete matrix with rank 10.
3. **MICE** [65]. Multiple imputation by chained equations. The algorithm fills the missing values iteratively until convergence. We use 10 nearest features and set the maximum iterations to 100.
4. **BRITS** [5]. BRITS has the similar bidirectional recurrent models as ours for time series imputation. It learns to impute only based on the time series features and does not consider the spatial information of the underlying graphs.
5. **rGAIN** [11]. A GAN based imputation model which is similar to SSGAN [50]. rGAIN can be regarded as an extension of GAIN [77] with bidirectional encoder and decoder.
6. **SAITS** [15]. SAITS is a self-attention based methods with a weighted combination of two diagonally-masked self-attention blocks, which is trained by a joint optimization approach on imputation and reconstruction.
7. **TimesNet** [66]. TimesNet transforms the 1D time series into 2D space and present the intraperiod- and interperiod-variations simultaneously. Its inception-block is able to discover multiple periods and capture temporal 2D-variations from the transformed data.
8. **GRIN** [11]. GRIN is a state-of-the-art model for MTS imputation with the relational information from a static and accurately known graph, which uses MPNN to build a spatiotemporal recurrent module and solves the problem in a bidirectional way.
9. **NET**^**3**^ [29]. NET^3^ is a recent work focusing on tensor time series learning and assumes that the tensor graphs are fixed and accurately known.

NTS imputation (i.e., Problem 1) also aims to solve the link prediction problem. We compare the performance of our method with following baselines:

1. **VGAE** [37]. Vanilla variational graph autoencoder is the first work that brings VAE to graph learning, and has competitive performance on link prediction task over static graphs.
2. **VGRNN** [24]. Variational graph recurrent neural networks extends VGAE to handle temporal information with the help of RNNs, and is a powerful baseline for the link prediction task on dynamic graphs.

#### 4.1.3 Metrics.

We use *mean absolute error (MAE), mean squared error (MSE) and mean relative error* (MRE) to evaluate the imputation performance of all models over missing features. For the link prediction task, we use the Frobenius norm as the metric since all the edges are weighted. All the experiments are run with 5 different random seeds and the results are presented as mean ± standard deviation (std).

### 4.2 Time Series Imputation Task

Empirical results from Table 3 and Table 4 demonstrate that the proposed PoGeVon outperforms all the baselines over the time series missing values prediction task in the NTS imputation problem. In particular, PoGeVon achieves more than 10% improvement on all the datasets compared with the best baselines. Especially, PoGeVon has significant improvements over all the baselines over COVID-19 dataset where other neural network based models except TimesNet have even worse performance than traditional time series imputation methods such as MF and MICE. It is worth noting that, although equipped with modules to handle topological information from graphs, GRIN and NET^3^ are less competitive than PoGeVon when the graph is constantly changing and contains missing edges. On the AQ36 dataset and the PeMS-SD dataset, they bear worse performance compared to BRITS and rGAIN, which do not leverage any topological information. PoGeVon outperforms BRITS and rGAIN by at least 12.92% and 10.55% on these two datasets respectively, which further indicates the effectiveness of our method. Although TimesNet is the strongest model over most of the datasets except AQ36, there still exists a large gap between its performance and PoGeVon even with much more parameters (triple number of parameters of PoGeVon). The main reason PoGeVon fluctuates (with a large std) on AQ36 dataset compared with traffic datasets is that AQ36 has fewer training samples (time steps), which brings more uncertainty for the model and results in larger differences of performances using different random seeds.

### 4.3 Link Prediction Task

VGAE and VGRNN were originally designed for link prediction over unweighted graphs. However, all the graphs are weighted in our NTS imputation settings, and thus, we modify these models correspondingly and apply the same Frobenius loss function we use in PoGeVon to train them. All the results are listed in Table 5. Both baselines have relatively worse performance compared to PoGeVon in all the datasets, and even using RNNs, VGRNN only gains minor improvement over VGAE. This indicates that both VGAE and VGRNN may not be able to handle the link prediction task over weighted dynamic graphs very well.

### 4.4 Ablation Studies

To evaluate the effectiveness of different components of our proposed method, we compare PoGeVon with following variants: (1) Replace RWR node position embeddings with the shortest path distance (SPD) based node embeddings by calculating the distance between each node with anchor nodes. (2) Replace RWR node position embeddings with the RWPE node position embeddings from [16]. (3) Replace RWR node position embeddings with the PGNN node position embeddings from [78]. (4) Remove the link prediction module in the 2^nd^ stage prediction. (5) Remove the self-attention module in the 3^rd^ stage prediction by replacing it with a linear layer. The results of the ablation study over AQ36 dataset are shown in Table 6. As we can see, the proposed method PoGeVon indeed performs the best which corroborates the necessity of all these components in the model.

#### 4.4.1 Sensitivity Analysis.

**Figure 4:**
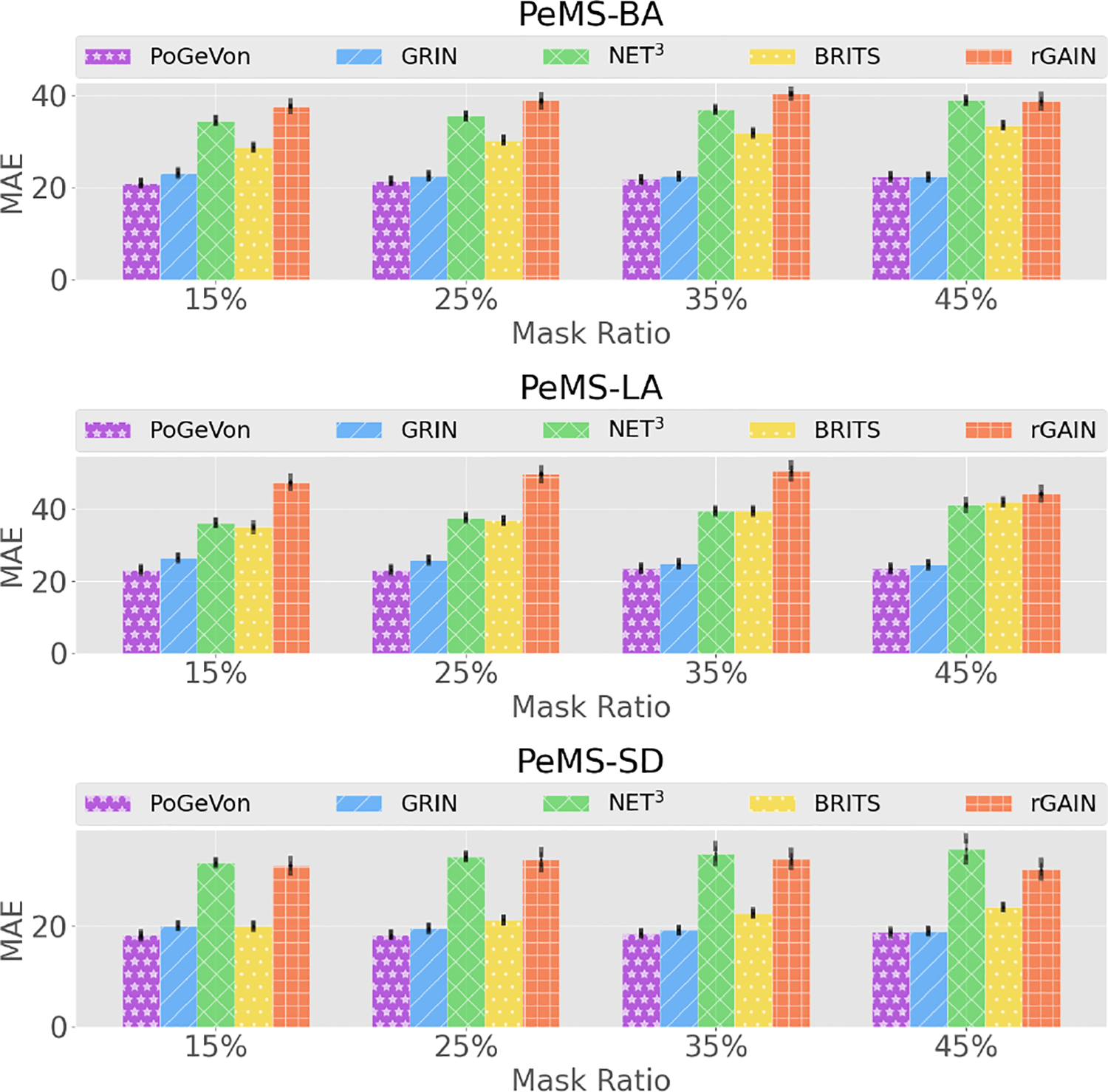
Sensitivity analysis for time series imputation with different masking rates on the traffic dataset. Lower is better. Best viewed in color.

We conduct sensitivity analysis to study the effect brought by increasing the masking rates. We consider the following mask rates: 15%, 25%, 35%, 45%. In order to keep a reasonable edge missing rate, for each edge with either end node being masked, they have 70% of chance being masked instead of using the setting from Eq. (20). The results are shown in Figure 4, in which the error bar demonstrates the standard deviation of MAE over 5 runs with different random seeds. The proposed PoGeVon consistently outperforms all the baselines in these settings which further demonstrates the effectiveness and robustness of our method.

## 5 RELATED WORK

In this section, we review the related works which can be categorized into two groups, including (1) multivariate time series imputation and (2) GNNs with relative position encodings.

### Multivariate Time Series Imputation.

In addition to traditional methods such as ARIMA [2] and K-Nearest Neighbors (KNN) [7], deep learning models are widely adopted in recent years to solve the MTS imputation problem. BRITS [5] is one of the most representative methods which uses bidirectional RNNs. There also exist a wide range of methods using deep generative models such as generative adversarial nets (GAN) [23] and VAE [35]. GAIN [77] is one of the earliest methods that use GAN to impute missing data, and later [46] applies GAN to the multivariate time series setting based on 2-stage imputation. E^2^GAN [47] is an end-to-end GAN and uses the noised compression and reconstruction strategy to generate more reasonable imputed values compared to previous works. SSGAN [50] proposes a novel method based on GAN to handle missing data in partially labeled time series data. VAE is used in GP-VAE [18] to solve the MTS imputation task with Gaussian process as the prior.

Other works handle MTS imputation problem from the perspective of spatial-temporal modeling, which takes the advantage of entities relations from the underlying graph. [4] is the first trial of using matrix factorization algorithm to recover missing values over MTS data with graph structures. More recently, GNNs have been used to capture the topological information in the MTS data. GRIN [11] proposes a novel bidirectional message passing RNN with a spatial decoder to handle both the spatial and temporal information. SPIN [48] uses sparse spatiotemporal attention to capture inter-node and intra-node information for predicting missing values in MTS. NET^3^ [29] generalizes the problem to tensor time series where multiple modes of relation dependencies exist in the time series. It introduces a tensor GCN [36] to handle the tensor graphs and then proposes a tensor RNN to incorporate the temporal dynamics. One common limitation of all these methods is that they either ignore the topological information from graph or assume the graph is fixed and accurately known.

### GNNs with Relative Position Encodings.

The expressive power of message-passing based GNNs has been proved to be bounded by 1-Weisfeiler-Lehman test (1-WL test) in [70]. Many follow-up works have been done to improve the expressive power of GNNs which go beyond 1-WL test, and position-aware graph neural networks (P-GNNs) [78] is one of them. P-GNNs randomly picks sets of anchor nodes and learn a non-linear distance-weighted aggregation scheme over these anchor sets for each node. This relative position encodings for nodes are proved to be more expressive than regular GNNs. Distance Encoding [40] uses graph-distance measures between nodes as extra features and proves that it can distinguish node sets in most regular graphs in which message-passing based GNNs would fail. [16] proposes a novel module for learnable structural and positional encodings (LSPE) along with GNNs and Transformers [61], which generates more expressive node embeddings. Recently, PEG [63] is introduced for imposing permutation equivariance and stability to position encodings, which uses separate channels for node features and position features. Compared with these existing methods, our proposed RWR-based position embedding could capture more topological information from the entire graph, as our analysis in Section 3.2.1 shows.

## 6 CONCLUSION

In this paper, we focus on solving networked time series imputation problem, which has two main challenges: (1) the graph is dynamic and missing edges exist, and (2) the node features time series contain missing values. To tackle these challenges, we propose PoGeVon, a novel VAE model utilizing specially designed RWR-based position embeddings in the encoder. For the decoder, we design a 3-stage predictions to impute missing values in both features and structures complementarily. Experiments on a variety of real-world datasets show that PoGeVon consistently outperforms strong baseline methods for the NTS imputation problem.

## Figures and Tables

**Table 1:** Symbols and Notations.

Symbol	Definition
𝓖	sequence of graphs
𝓐	tensor of graph adjacency sequence
𝓧	tensor of multivariate time series
𝓜	mask tensor of 𝓧
𝓡	tensor of node position embeddings
$G_{t}$	graph at time step t
${\tilde{G}}_{t}$	observed graph at time step t
$\tilde{𝓖}$	observed sequence of graphs
$\tilde{𝓐}$	observed tensor of graph adjacency sequence
$\tilde{𝓧}$	observed multivariate time series
$A_{t}$	adjacency matrix at time t
$X_{t}$	node feature matrix at time t
$M_{t}$	mask matrix at time t
$R_{t}$	RWR position matrix at time t
$r_{t,i}$	RWR position score of node i at time t
$e_{i}$	one-hot restart vector with value 1 at index i
$D={\text{diag}}^{2}(d)$	diagonal matrix of the degree vector d
$A^{⊤}$	transpose of A
Z	latent node embedding matrix of VAE
H(X)	entropy of random variable X
I(X;Y)	mutual information between X and Y
T	length of time series
N	number of nodes
D	number of features
i,j,u,v	indices of nodes
c	restart probability in RWR
z	latent representations of VAE
θ,γ,ϕ	parameters of neural networks
$\|\cdot {\|}_{F}$	Frobenius norm
⊙	Hadamard product

**Table 2:** Statistics of the datasets. Entity numbers of PeMS* datasets refer to the original number of sensors/stations in the corresponding dataset and only part of them are used to build the graphs.

Dataset	# of entity	# of nodes	average # of edges	time length
COVID-19	50	50	1344.75	346
AQ36	36	36	341.57	8759
PeMS-BA	1632	64	675.45	25920
PeMS-LA	2383	64	1095.54	25920
PeMS-SD	674	64	1295.11	25920

**Table 3:** Performance comparison over COVID-19 and AQ36 datasets. Smaller is better.

		COVID-19			AQ36	
Models	MAE	MSE	MRE	MAE	MSE	MRE
Mean	3.081 ± 0.000	10.707 ± 0.000	0.284 ± 0.000	62.299 ± 0.000	6525.709 ± 0.000	0.835 ± 0.000
MF	0.276 ± 0.026	0.165 ± 0.025	0.026 ± 0.002	39.582 ± 0.189	4545.596 ± 61.411	0.531 ± 0.002
MICE	0.077 ± 0.005	0.013 ± 0.002	0.007 ± 0.000	38.889 ± 0.268	4314.435 ± 20.617	0.521 ± 0.003
BRITS	0.386 ± 0.006	0.293 ± 0.009	0.036 ± 0.001	23.393 ± 0.802	1276.226 ± 102.916	0.314 ± 0.011
rGAIN	0.579 ± 0.069	0.571 ± 0.106	0.055 ± 0.006	25.032 ± 1.426	1358.134 ± 152.361	0.335 ± 0.019
SAITS	0.466 ± 0.010	0.366 ± 0.019	0.043 ± 0.001	51.097 ± 0.625	5026.475 ± 75.120	0.685 ± 0.008
TimesNet	0.028 ± 0.002	0.002 ± 0.000	0.003 ± 0.000	40.700 ± 0.278	3383.554 ± 49.499	0.545 ± 0.004
GRIN	0.319 ± 0.038	0.165 ± 0.040	0.029 ± 0.004	29.420 ± 0.231	2050.726 ± 56.028	0.394 ± 0.003
NET^3^	0.547 ± 0.004	0.682 ± 0.006	0.051 ± 0.000	34.755 ± 0.497	2473.718 ± 37.461	0.466 ± 0.007
PoGeVon	**0.007** ± **0.001**	**0.000** ± **0.000**	**0.001** ± **0.000**	**19.494** ± **1.101**	**1213.474** ± **125.529**	**0.261** ± **0.015**

**Table 4:** Performance comparison over PeMS-BA, PeMS-LA and PeMS-SD datasets. Smaller is better.

		PeMS-BA			PeMS-LA			PeMS-SD	
Models	MAE	MSE	MRE	MAE	MSE	MRE	MAE	MSE	MRE
Mean	192.047 ± 0.000	47504.159 ± 0.000	0.474 ± 0.000	216.681 ± 0.000	62664.657 ± 0.000	0.406 ± 0.000	208.192 ± 0.000	55780.002 ± 0.000	0.529 ± 0.000
MF	57.265 ± 1.148	8091.407 ± 185.123	0.141 ± 0.003	77.339 ± 0.699	15202.678 ± 156.348	0.145 ± 0.001	45.811 ± 0.318	6044.345 ± 72.976	0.117 ± 0.001
MICE	50.861 ± 0.765	6724.148 ± 109.829	0.126 ± 0.002	64.018 ± 1.015	10822.355 ± 405.410	0.120 ± 0.002	38.978 ± 1.036	4771.186 ± 92.335	0.100 ± 0.003
BRITS	30.274 ± 0.095	2942.411 ± 16.511	0.075 ± 0.000	36.921 ± 0.133	3681.595 ± 21.635	0.069 ± 0.000	21.232 ± 0.059	1563.234 ± 28.309	0.054 ± 0.000
rGAIN	38.862 ± 0.752	3422.914 ± 61.281	0.096 ± 0.002	49.611 ± 1.083	5533.964 ± 234.335	0.093 ± 0.002	33.212 ± 1.475	2341.466 ± 98.314	0.085 ± 0.004
SAITS	46.567 ± 0.530	5412.574 ± 161.132	0.115 ± 0.001	61.896 ± 0.892	10998.854 ± 204.345	0.116 ± 0.002	34.117 ± 0.886	4101.397 ± 152.141	0.087 ± 0.002
TimesNet	25.859 ± 0.115	1676.843 ± 16.144	0.064 ± 0.000	27.452 ± 0.114	2058.227 ± 6.213	0.052 ± 0.000	21.583 ± 0.085	1284.300 ± 21.839	0.055 ± 0.000
GRIN	30.057 ± 1.073	1922.072 ± 74.327	0.074 ± 0.003	47.835 ± 2.059	4561.512 ± 298.533	0.090 ± 0.004	41.001 ± 1.543	3000.012 ± 201.018	0.105 ± 0.004
NET^3^	35.671 ± 0.111	2735.574 ± 6.138	0.009 ± 0.000	37.652 ± 0.113	3416.784 ± 6.765	0.071 ± 0.000	34.111 ± 0.184	2487.581 ± 9.798	0.087 ± 0.000
PoGeVon	**22.194** ± **0.046**	**1248.681** ± **4.297**	**0.055** ± **0.000**	**23.905** ± **0.245**	**1714.962** ± **31.035**	**0.045** ± **0.000**	**18.990** ± **0.112**	**951.559** ± **8.264**	**0.048** ± **0.000**

**Table 5:** Performance comparison of the link prediction task in NTS imputation. Smaller is better.

Models	AQ36	PeMS-BA	PeMS-LA	PeMS-SD
VGAE	134.42 ± 0.11	431.94 ± 2.31	404.17 ± 1.76	399.78 ± 1.29
VGRNN	133.92 ± 0.29	428.82 ± 0.01	402.30 ± 0.90	398.81 ± 0.01
PoGeVon	**95.42 ± 1.80**	**148.44 ± 0.31**	**168.05 ± 0.31**	**185.86 ± 0.15**

**Table 6:** Ablation study of PoGeVon over AQ36 dataset on time series feature imputation. Smaller is better.

Models	MAE	MSE	MRE
PoGeVon	**19.49 ± 1.10**	**1213.47 ± 125.53**	**0.26 ± 0.02**
change RWR to SPD	21.98 ± 1.55	1309.55 ± 199.24	0.33 ± 0.02
change RWR to RWPE Embeddings	23.75 ±0.85	1597.67 ±210.77	0.32 ±0.01
change RWR to PGNN Embeddings	24.46 ±2.59	1625.19 ±393.25	0.33 ±0.04
w/o link prediction in 2^nd^ stage	28.71 ± 3.38	2130.46 ± 417.45	0.38 ± 0.05
w/o self-attention in 3^rd^ stage	23.40 ± 1.00	1576.06 ± 194.45	0.31 ± 0.01

**Figure 5:**
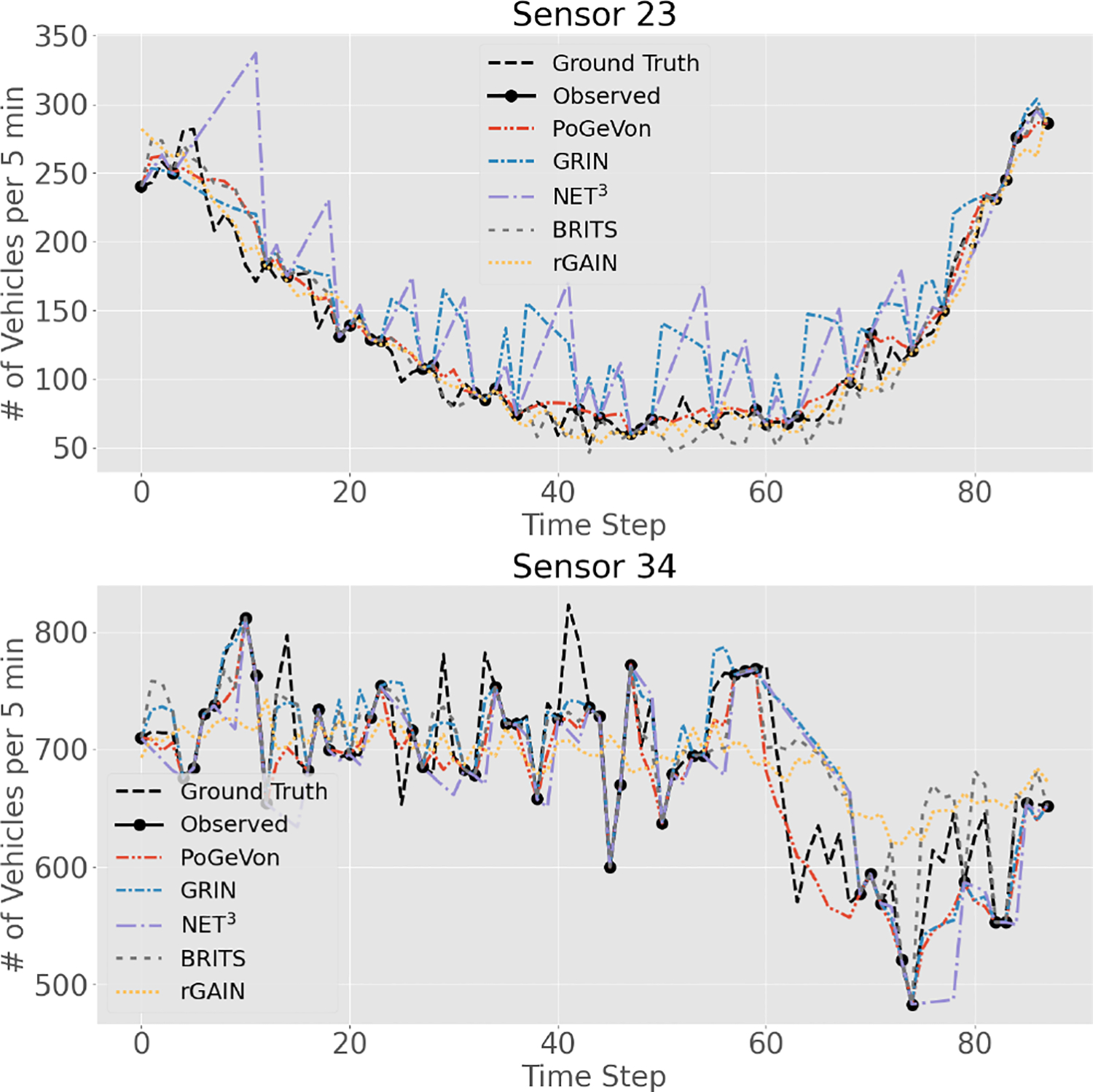
Different models’ predictions of traffic flow in sensor 23 and 34 from PeMS-LA dataset. Best viewed in color.

## CCS CONCEPTS

Information systems → Data mining; • Computing methodologies → Machine learning.

## Footnotes

1 In some research works [39], NTS is also named as *network time series*.

## A APPENDIX

In the appendix, we present the additional details of PoGeVon including

- Proofs of Proposition 3.1 and Theorem 3.2 in Section A.1 and Section A.2 respectively.
- Additional details of components in PoGeVon is introduced in Section A.3.
- Reproducibility and parameter settings of baselines and the proposed PoGeVon are listed in Section A.4.
- Additional experiments such as visualization for prediction, ablation study over self-attention in PoGeVon and ablation study over RWR restart probability c in PoGeVon are given in Section A.5.
- Section A. 6 discusses the limitations of our implementations of PoGeVon and propose some potential future works based on NTS imputation.

### A.1 Proof Proposition 3.1

We prove Proposition 3.1 by analyzing the properties of RWR.

Proposition. *Random walk with restarts (RWR) captures information from close neighbors (local) and long-distance neighbors (global) in graph learning*.

Proof. Based on Eq. (6), the closed form solution for RWR can be derived as: $r_{i}=c\cdot (I-(1-c)\cdot \overset{ˆ}{A}{)}^{-1}e_{i}$, where I is the identity matrix. We could also solve this equation by power iterations: $(I\;-(1-c)\cdot \overset{ˆ}{A}{)}^{-1}\approx \sum_{k=0}^{\infty }((1-c)\cdot \overset{ˆ}{A}{)}^{k}$. First of all, as the power term k goes to infinity, the position embedding R can indeed capture *global* information of graph. Second, the restart probability c ensures nodes close to anchor nodes have larger values than those farther away, which encodes the local information of graph.

*Remark*. Proposition 3.1 holds with the assumption that the graph is connected. When graph is not connected, with proper choice of a set of anchor nodes that cover all the connected components, RWR is able to *global* information within each components rather than the entire graph. An alternative to generate node position embeddings is using RWR from each node similar to [16] to get the landing probability of a node to itself for multiple steps. However, this usually increases the complexity when having a large graph and still faces similar issues as ours when graph is less connected. □

### A.2 Proof of Theorem 3.2

To prove Theorem 3.2, we first introduce the following proposition.

Proposition A.1. For any random variables A,B, and C, the following inequality of the mutual information I(·;.) *holds* [54]*:*

(21)
I(A,B;C)≥I(A;C)

Proof. Based on the chain rule of mutual information, we have:

I(A,B;C)=H(A,B)−H(A,B∣C)=H(A)+H(B∣A)−H(A∣C)−H(B∣A,C)=I(A;C)+I(B;C∣A)

where H(A) is the marginal entropy, H(A∣C) is the conditional entropy and H(A,B) is the joint entropy. Since the mutual information I(B;C∣A)≥0, we can conclude that I(A,B;C)≥I(A;C). □

Now we can prove Theorem 3.2 as:

Theorem. *Given a temporal graph* 𝓖, *TGN with RWR-based node position embeddings* $g_{\theta }$ *has more expressive power than regular TGN* $f_{\theta }$ in node representation learning: D(g(u),g(v))≥D(f(u),f(v)) *where* D(⋅,⋅) *measures the expressiveness by counting the distinguishable node pairs* (u,v) *in* 𝓖 *based on node representations*.

Proof. It is natural to see that $g_{\theta }$ has at least same expressive power as $f_{\theta }$ since we add additional information with the positional embeddings for each node. By setting all the parameters of $g_{\theta }$ that handle such positional embeddings to zero, we will have a regular TGN model same as $f_{\theta }$.

To prove that why the additional information brought by node position embeddings is useful for node representation learning, we provide following analysis with the help of Proposition A.1. Regular TGN only encodes topologically local information within q-hop neighbors and q usually is a small number because of the oversmoothing problem [41], we denote the random variable for $f_{\theta }$’s node representations as $X_{\text{local}}$. Based on Proposition 3.1, we know that the random variable for $g_{\theta }$’s node representations $X_{\text{local}+\text{global}}$ follows the joint distribution of both local and global topological information. The objective of a node representation learning task over graph 𝓖 can be denoted as max I(X;Y) where Y is the random variable follows label distributions [78]. This derivation can be obtained from Information Bottleneck we discussed in Section 3.1 without the constraints term. Therefore, based on Proposition A.1, we have $I\left(X_{\text{local}+\text{global}};Y\right)\geq I\left(X_{\text{local}};Y\right)$ which denotes that $g_{\theta }$ has more expressive power than $f_{\theta }$. □

### A.3 Additional Details over PoGeVon

#### A.3.1 Details of message-passing neural network.

The message-passing neural network (MPNN) used in PoGeVon is defined as:

(22)
$$\text{MPNN}\left(F_{u},F_{m},d_{t,i},A\right)=F_{u}\left(d_{t,i},\sum_{j\in 𝓝(i)}F_{m}\left(h_{t,i},d_{t,j},e_{i,j}\right)\right)$$

where $F_{u}$ and $F_{m}$ are update and message functions with learnable parameters, $d_{t,i}$ is the node representation for node i at time step $t,\;e_{i,j}$ is the edge weight between node i and j, and 𝓝(i) represents node i ‘s neighbors. The two-layer MPNNs with skip connection used in PoGeVon can be defined as:

(23)
$$H_{1,t}=\text{MPNN}\left(F_{u}^{1},F_{m}^{1},U_{t},A_{t}^{\text{out}}\right)\;H_{2,t}=\text{MPNN}\left(F_{u}^{2},F_{m}^{2},H_{t,1},A_{t}^{\text{out}}\right)\;H_{t}^{\text{graph}}=H_{1,t}⊕H_{2,t}$$

where ⊕ is the element-wise addition.

#### A.3.2 Details of self-attention.

The self-attention module is defined as:

(24)
$$\text{Attn}\left(h_{t,i}\right)=\sum_{j\in 𝓝(i)}\text{softmax}\left(\frac{Q_{i}K_{j}^{T}}{\sqrt{d}}\right)V_{j}\;\text{and}\;Q_{i}=h_{t,i}W_{Q},K_{j}=h_{t,j}W_{K},V_{j}=h_{t,j}W_{V}$$

where 𝓝(i) is the neighbor of node i,d is the hidden dimension and $W_{Q},W_{K},W_{V}$ are learnable parameters.

### A.4 Reproducibility

We introduce the detailed parameter settings of our models as well as baselines in this subsection. For PoGeVon, the restart probability c of RWR for position embeddings is set to the commonly used 0.15, and we picked the number of anchor nodes as $L={\text{log}}_{2}\;(N)$. We set the hidden dimension to be 64, β to be 0.2 and γ to be 0.01.

All the experiments are based on codes from a open source library^2^ [56] and those provided by corresponding authors. We modify their implementations for the NTS imputation problem and the details of parameter settings are listed as follows: for the parameters for each baseline model in the time series feature imputation, we refer to previous works for their settings [5, 11]. For BRITS^3^, we use the same hidden dimension as [5] for AQ36 dataset, and for the traffic datasets, the hidden size is set to 256 which is aligned with the setting used in PeMS data in [11]. For rGAIN, we follow the exact same setting as in [11, 48] in which we use 64 as the hidden size for AQ36 dataset and 256 for traffic datasets. For SAITS and TimesNet, we follow the exact parameter settings in their papers [15, 66]. For GRIN^4^, we use the same hidden size for AQ36 as [11], while using 80 for the traffic datasets since they are much larger than AQ36. As for the hidden dimension of NET^3 5^, we use 128 for AQ36 dataset and 256 for traffic datasets. For COVID-19 dataset, we use the same parameters settings of traffic datasets for all the models.

For VGAE and VGRNN in the link prediction task in NTS imputation, we use hidden size 256/128 for the AQ36 and 320/150 for the traffic datasets respectively.

We train all the models using PyTorch [53] with Adam optimizer [34], learning rates are set to be 0.001/0.01 for time series feature imputation and link prediction baselines respectively with cosine annealing scheduler [45] to adjust the values dynamically. The batch sizes are all set to 32 and we use validation dataset for early stopping.

### A.5 Additional Experiments

#### A.5.1 Visualization of Prediction.

The prediction results of different selected baselines over PeMS-LA with 50% missing rates over test data can be found in Figure 5. It is clear that PoGeVon can achieve better predictions results compared with other baselines. In particular, GRIN and NET^3^ sometimes suffer a lot from fluctuations due to the missing edges in the NTS data, which result in poor performance compared to our proposed PoGeVon. Besides, we can see that PoGeVon can have finer predictions compared with all the baselines when there exist abrupt change of data values which also has high missing rates (e.g., data from time step 60 to 65 in the figure of prediction of sensor 34).

#### A.5.2 Ablations on self-attention in PoGeVon.

We conduct ablation study over the self-attention module in PoGeVon by changing it to attention mechanism used in Linformer and Reformer respectively. The results of time series imputation over COVID-19 and AQ36 datasets are shown in Tables 7 and 8 respectively. It is clear that replacing vanilla self-attention with Linformer self-attention as well as LSH self-attention from Reformer can still achieve better results on COVID-19 with the strongest baseline MICE. They can also have better or comparable results over AQ36 compared with the strongest baseline BRITS as well.

**Table 7:** Ablation study of self-attention in PoGeVon over COVID-19 datasets on time series feature imputation. Smaller is better.

Model	MAE	MSE	MRE
MICE	0.077 ±0.005	0.013 ±0.002	0.007 ±0.000
PoGeVon	**0.007 ±0.001**	**0.000 ±0.000**	**0.001 ±0.000**
PoGeVon with Linformer self-attention	0.012 ±0.004	0.001 ±0.000	0.001±0.000
PoGeVon with LSH self-attention	0.009 ±0.004	0.000 ±0.000	0.001 ±0.000

**Table 8:** Ablation study of self-attention in PoGeVon over AQ36 datasets on time series feature imputation. Smaller is better.

Model	MAE	MSE	MRE
BRITS	23.39 ±0.80	1276.23 ±102.92	0.31 ±0.011
PoGeVon	**19.49 ±1.10**	**1213.47 ±125.53**	**0.26 ±0.02**
PoGeVon with Linformer self-attention	21.49 ±1.40	1333.61 ±168.71	0.28 ±0.02
PoGeVon with LSH self-attention	23.69 ±1.35	1422.27 ±123.62	0.32 ±0.02

#### A.5.3 Ablations on RWR Restart Probability in PoGeVon.

We have also conducted ablation studies on the RWR restart probability for our position node embeddings in PoGeVon. In literature, the restart probability is often set to be a small number (e.g., 0.15). A high restart probability makes RWR-based node embeddings near anchor nodes be more similar to each other which increases the local information while diminishes the global information. A low restart probability sometimes results in a sparse matrix because of the deadend nodes of graphs and RWR algorithm will degenerate to a vanilla PageRank/Random Walk equations. The experiment results on this aspect by setting the restart probability to different levels: 0.1, 0.2, 0.4 and 0.8 are shown in the Table 9. Based on the experiment results, we do observe performance drop of PoGeVonwhen choosing less effective restart probability for RWR-based node position embeddings. However, PoGeVondemonstrates certain stability and can still outperform other baseline models.

**Table 9:** Ablation study of RWR restart probability c in PoGeVon over COVID-19 datasets on time series feature imputation. Smaller is better.

Model	MAE	MSE	MRE
PoGeVon w. c=0.1	0.00734 ±0.00158	0.00017 ±0.00012	0.00068 ±0.00015
PoGeVon w. c=0.2	0.00785 ±0.00222	0.00021 ±0.00016	0.00073 ±0.00021
PoGeVon w. c=0.4	0.00739 ±0.00154	0.00015 ±0.00012	0.00064 ±0.00014
PoGeVon w. c=0.8	0.00737 ±0.00167	0.00014 ±0.00007	0.00066 ±0.00016
PoGeVon	**0.00690 ±0.00085**	**0.00013 ±0.00007**	**0.00064 ±0.00008**

### A.6 Limitations and Future Works

One limitation of the proposed PoGeVon model lies in its quadratic complexity $O\left(N^{2}\right)$ due to the self-attention module. As we have discussed in Section 3.5, we can reduce this complexity to either O(N) by Linformer [64] or O(NlogN) by Reformer [38]. Another limitation lies in the potential negative transfer effect, which might happen when negative correlation exists between the time series of adjacent node pairs. Under such circumstances, directly applying multi-task learning framework in PoGeVon might hurt the performance of NTS imputation. A possible solution is to resort to GNNs designed for graphs with heterophily [9, 44, 81, 83] in the decoder of the proposed PoGeVon. There are several interesting aspects that are worth future study, including (1) generalizing the proposed PoGeVon for detecting anomalies on NTS, forecasting NTS [30, 67] and assisting temporal graphs analysis [19, 21]; (2) applying it to temporal knowledge graph completion [27] and alignment [72] as well as dynamic recommendations [42].
